# Retrospective study of patients receiving long-term mechanical ventilation

**DOI:** 10.1186/cc14332

**Published:** 2015-03-16

**Authors:** I Dunwoody, B Hopwood, R Sinclair

**Affiliations:** 1 Royal Cornwall Hospital, Truro, UK

## Introduction

This study analysed the practice of clinicians managing patients requiring long-term mechanical ventilation in the critical care unit (CCU) of the Royal Cornwall Hospital, Truro (RCHT), comparing outcomes of primary tracheostomy (TR) with trial of extubation (TOE).

## Methods

All 89 inpatients on the CCU who received mechanical ventilation continuously for 7 days or more between October 2012 and December 2013 were initially included. Forty patients who were intubated prior to arrival at RCHT, had incomplete notes, or were extubated during end-of-life care were excluded. Patients were divided into groups by first airway intervention; 31 TOE, 18 TR.

## Results

A total 52% (16/31) of patients had TOE, required no other airway intervention and survived to discharge from hospital, compared with 72% (13/18) of TR patients. Four patients from each group failed the first intervention and died prior to a second intervention. In total, 8/11 patients who had a second intervention after failed TOE survived to discharge from hospital. One patient had a second TR but died before discharge. This gave an in-hospital mortality rate of 19% for the TOE group and 28% for the TR group. TOE was performed earlier, all 31 on days 7 to 15. TR was performed later; 14/18 on days 7 to 15, and 4/18 on days 17 to 23. Early TR was more successful; 11/11 survived to discharge without a second intervention who had TR on days 7 to 12, compared with 29% (2/7) after day 12. TOE was more successful when performed later; 64% (7/11) survived to discharge without a second airway intervention when TOE was after day 10, 45% (9/20) between days 7 and 10. After first failed TOE, four patients had a successful second TOE; all four survived to discharge resulting in a median CCU stay of 29 days and median hospital stay of 39 days (excluding prior to CCU admission). Seven patients had TR after the first failed TOE, five survived to discharge from the CCU and four to discharge from the hospital. This group had shorter median stays in both the CCU (27 days) and hospital (32 days). Overall, the median duration of time ventilated, in the CCU, and in hospital was shorter for the TOE group; 13, 17 and 24 days respectively, compared with 22, 27.5 and 34 days for the TR group.

## Conclusion

TOE is more common and is associated with shorter time spent ventilated, in the CCU and in hospital than TR. It is also associated with a lower in-hospital mortality rate. TOE is more successful when performed after day 10; TR is more successful when performed before day 13. After failed TOE, a second TOE is associated with longer time in hospital but a better mortality rate than secondary tracheostomy.

